# Bibliometric and visualization analyses of cancer-related fatigue research published worldwide from 2001 to 2023

**DOI:** 10.3389/fonc.2024.1338325

**Published:** 2024-04-30

**Authors:** Peijin Li, Qian Wang, Li Feng, Zhiguo Ding, Weijing Fan

**Affiliations:** ^1^ Department of Traditional Chinese Medicine, National Cancer Center/National Clinical Research Center for Cancer/Cancer Hospital, Chinese Academy for Medical Sciences and Peking Union Medical College, Beijing, China; ^2^ Department of Oncology, Dongzhimen Hospital of Beijing University of Chinese Medicine, Beijing, China; ^3^ Department of Thyropathy, Sunsimiao Hospital of Beijing University of Chinese Medicine, Tongchuan, Shanxi, China; ^4^ Department of Thyropathy, Dongzhimen Hospital of Beijing University of Chinese Medicine, Beijing, China; ^5^ Department of Vascular Surgery, Shuguang Hospital Affiliated to Shanghai University of Traditional Chinese Medicine, Shanghai, China

**Keywords:** cancer-related fatigue, bibliometric analysis, CiteSpace, inflammation, hypoxia, exercise

## Abstract

**Objective:**

Cancer seriously endangers human health and represents a global public health issue. Cancer-related fatigue (CRF) is a distressing and persistent sense of exhaustion caused by cancer or cancer treatment, widely prevalent among cancer patients. This study aims to summarize emerging trends and provide directions for future research of CRF through bibliometric and visualization analyses.

**Methods:**

A systematic search in the Web of Science Core Collection database from 2001-01-01 to 2023-05-18 were conducted. Only reviews and articles written in English were considered. CiteSpace and the R were used for bibliometric and visualization analyses.

**Results:**

The analysis revealed that 2,566 studies on CRF have been published by 1,041 institutions in 70 countries so far. The number of articles published and cited annually have been steadily increasing. Eduardo Bruera published the most articles, and Julienne E Bower is the most co-cited author. The University of Texas System is the leading institution in cancer-related fatigue research. The United States and China have the largest number of publications. Supportive Care in Cancer published the most articles, and Journal of Clinical Oncology is the most co-cited journal. “Comparison of Pharmaceutical, Psychological, and Exercise Treatments for Cancer-Related Fatigue: A Meta-analysis”, authored by Mustian KM et al. and published in JAMA Oncology was the most co-cited document. Keyword analysis indicated that research focus had shifted from “epoetin alpha” and “anemia” to “risk factors”, “systematic review”, “acupuncture”, “anxiety”, “traditional Chinese medicine” and “guidelines”.

**Conclusion:**

In conclusion, this analysis provides comprehensive research trends and knowledge network maps of CRF. Clinical physicians should concurrently focus on the anemia, insomnia, anxiety, and depression status of patients when assessing or managing CRF. Improvements in related risk factors also contribute to alleviating fatigue. Furthermore, it is essential to pay attention to authoritative CRF guidelines. Acupuncture and traditional Chinese medicine also have therapeutic potential, which merits further investigation. Researchers should draw attention to the crucial roles of inflammation, hypoxia, and mitochondrial dysfunction, which could be the frontiers.

## 1 Introduction

Cancer-related fatigue (CRF) is a subjective feeling of tiredness or exhaustion associated with cancer or cancer treatment, encompassing physical, emotional, and/or cognitive aspects (1). In a meta-analysis of 144,813 participants conducted in 2020, the estimated diagnostic yield for CRF was 52% (2). Up to 80% of patients treated with chemotherapy and/or radiotherapy experience CRF (3). Chemotherapy and radiotherapy are the main factors that contribute to CRF. Other therapeutic modalities, such as targeted therapy, immunotherapy, and surgical treatments, o cancer itself, are also associated with CRF.

Cancer survivors may experience fatigue for months, years, or even up to a decade (4). CRF is more distressing and long-lasting than general fatigue and cannot be alleviated by resting. It often causes patients with cancer unbearable suffering, severely affects quality of life, disrupts daily functioning, interferes with subsequent therapies, and influences prognosis. Therefore, alleviating CRF is crucial for improving the overall quality of life of patients with cancer.

Since the 20th century, many studies have revealed the occurrence of fatigue symptom in cancer patients. Consequently, studies have been conducted to elucidate the assessment, treatment, and possible mechanisms of CRF, providing new perspectives and hotspots in the field of cancer research. Nevertheless, effective measures for reducing CRF are still limited, which are primarily non-pharmacological treatments, such as exercise and yoga. This is partly due to the obscure mechanisms underlying the occurrence of CRF. Currently, the proposed possible hypotheses for CRF include the dysregulation of inflammatory cytokines and dysfunction of skeletal muscles and mitochondria. It is important for physicians and researchers to understand the progression and characteristics of research related to CRF, which can facilitate the identification of future research directions and more efficient approaches to treating CRF.

Bibliometric analysis is an emerging analytical technique that is used to summarize advances in a certain field, reveal research hotspots, and quantitatively outline the contributions of authors, journals, institutions, and countries (5). However, no bibliometric or visualization analyses of studies on CRF have been conducted to date. CiteSpace (6) is a bibliometric tool that is commonly used to visualize information extracted from articles. The aim of this study was to conduct bibliometric and visualization analyses of CRF research published in the Web of Science Core Collection (WoSCC) database from 2001 to 2023, to summarize emerging trends and provide directions for future research.

## 2 Methods

### 2.1 Search strategy and data acquisition

**Figure 1:**
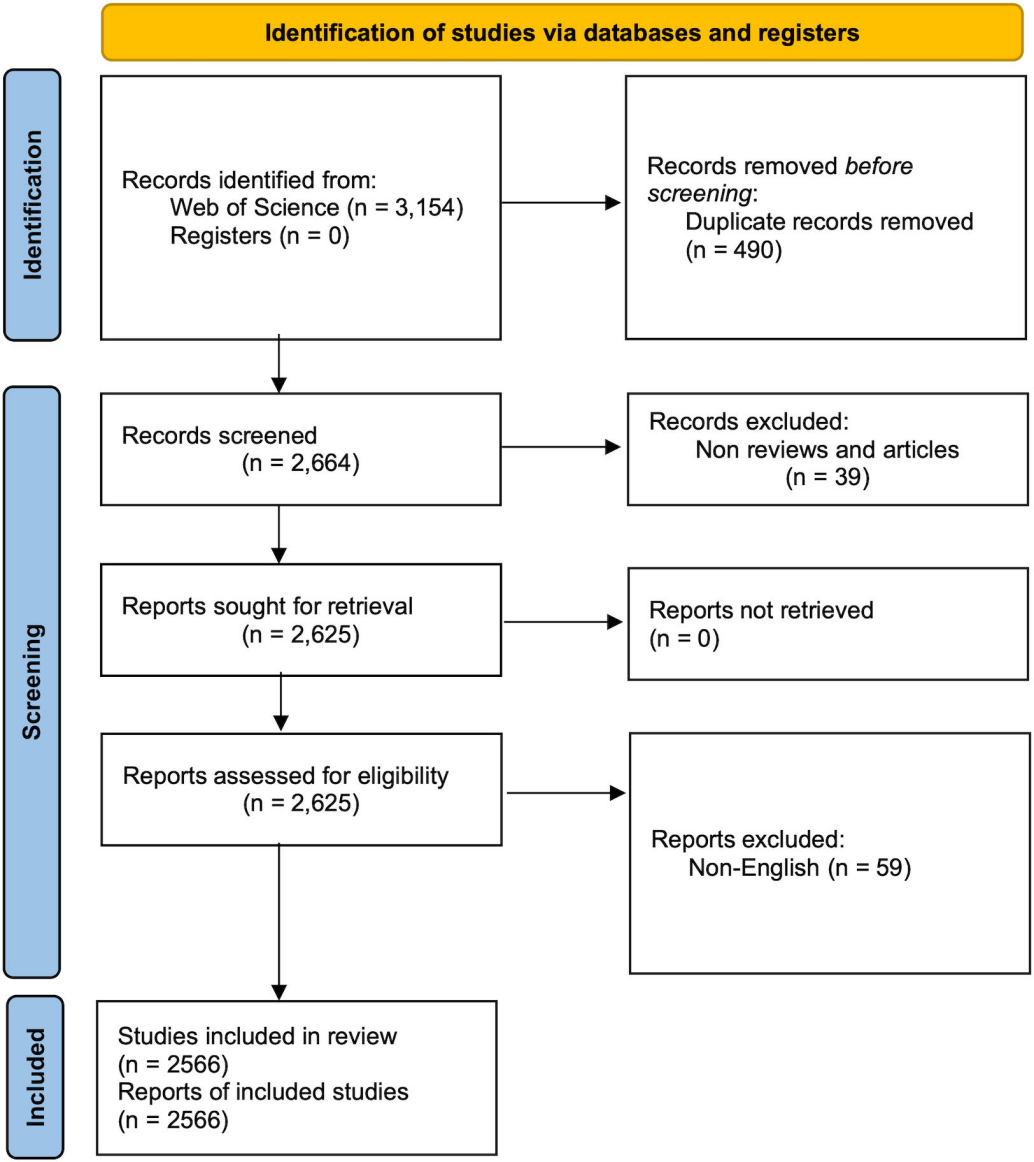
Flowchart of the retrieval process.

Two investigators (PJL and QW) conducted a systematic search in the WoSCC database, covering the period from January 1st, 2001 to May 18th, 2023. In cases of disagreement, a third reviewer (ZGD) provided the final decision. The search queries used were TS = (cancer-related fatigue), and the publication date range was set from 2001-01-01 to 2023-05-18. The search was limited to the Social Sciences Citation Index (SSCI) and Science Citation Index Expanded (SCI-EXPANDED) editions. Only reviews and articles written in English were considered, while other article types such as conference abstracts and editorials were excluded. This study was performed based on the Preferred Reporting Items for Systematic Reviews and Meta-analyses (PRISMA) guidelines (7) (**Supplementary Table 1**). The systematic search yielded 3,154 records, and 2,566 studies were included in the final analysis (**Figure 1**). All relevant publications were downloaded on May 18th, 2023. The results were stored in download_results.txt format, and the content included “full record and cited references.”

### 2.2 Data analysis

All data were imported into CiteSpace 6.2.R3 and duplicate entries were eliminated. The publication year was analyzed using WoSCC. The collaboration among authors, institutions, and countries, as well as the co-citation of references and journals, and the co-occurrence of keywords were visualized. The R package “bibliometrix” (8) was employed to analyze the geographical distribution of countries. The findings were presented in figures and tables using techniques such as time slicing, thresholding, and pruning. We set the time span as 2001.01–2023.12 and the years per slice as 1, and chose the suitable pruning methods (minimum spanning tree, pathfinder, or no) according to the analysis results. The size of nodes in the various network maps was indicative of the frequency of co-occurrence or co-citation, while the color denoted the year. The citation bursts of references and keywords were analyzed to find the emerging themes in the CRF field. In the journal co-citation analysis, we merged the same journals (“cancer” and “cancer-am cancer soc”) to an alias list. The synonyms in keyword analysis were also merged, including “cancer-related fatigue” and “cancer related fatigue,” “cancer survivors” and “survivors,” etc.

## 3 Results

### 3.1 Annual distribution of publications

**Figure 2:**
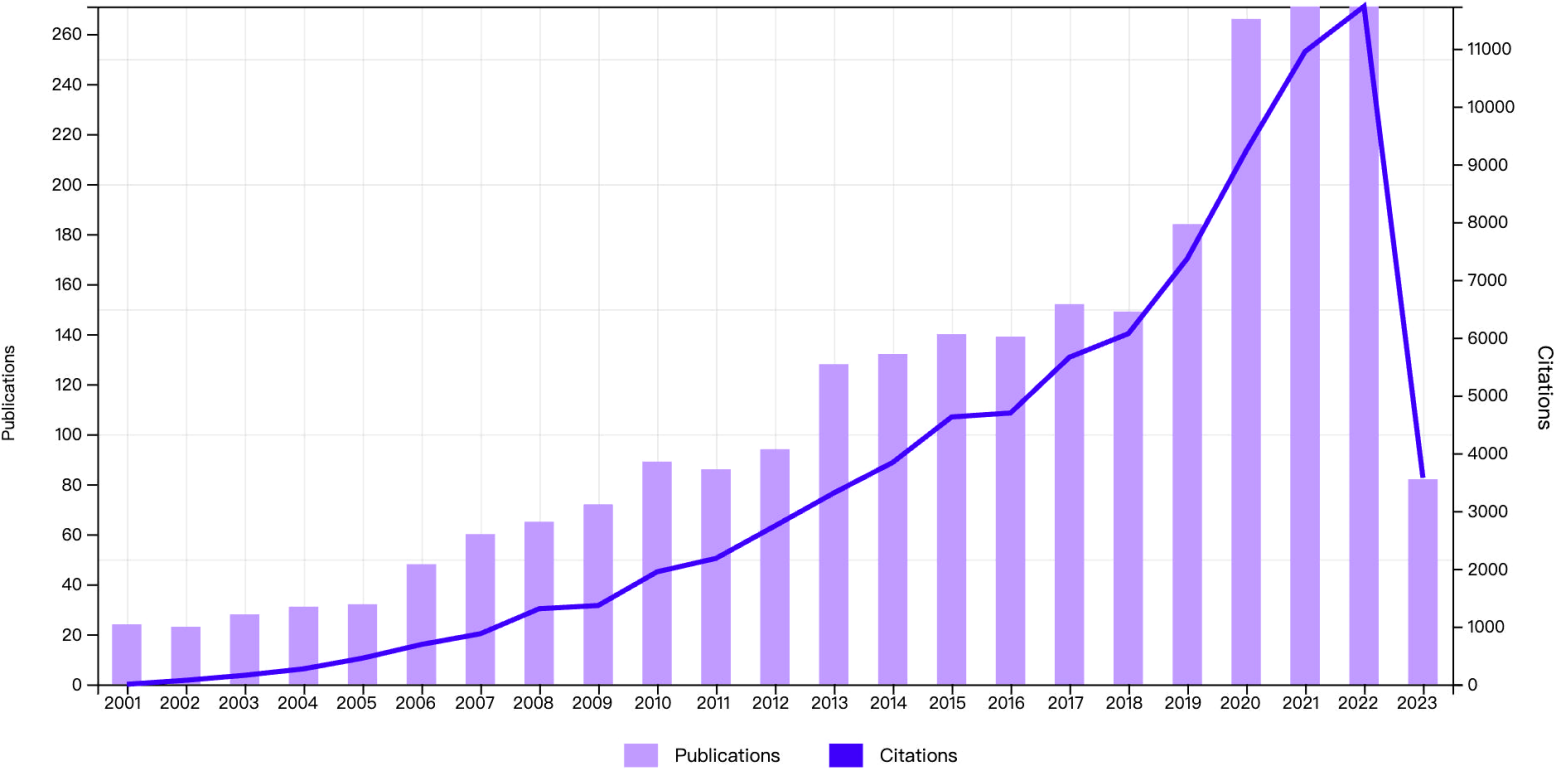
Annual distribution of publications. The bar graph represents the number of publications and the line graph represents the number of citations.

2566 articles and review articles pertaining to CRF were published from January 2001 to May 2023. As depicted in **Figure 2**, there has been a stable growth trend in the number of annual publications and citations since 2001, as heightened interests among scholars in this domain. Notably, the most number of publications reached 271 in 2021 and 2022, and the highest increasing number reached 82 from 2019 to 2020. The citations ranged from 69 in 2002 to a substantial 11,731 in 2022, reflecting increasing attention to the CRF field.

### 3.2 Analysis of co-authorship and co-cited authors

**Figure 3:**
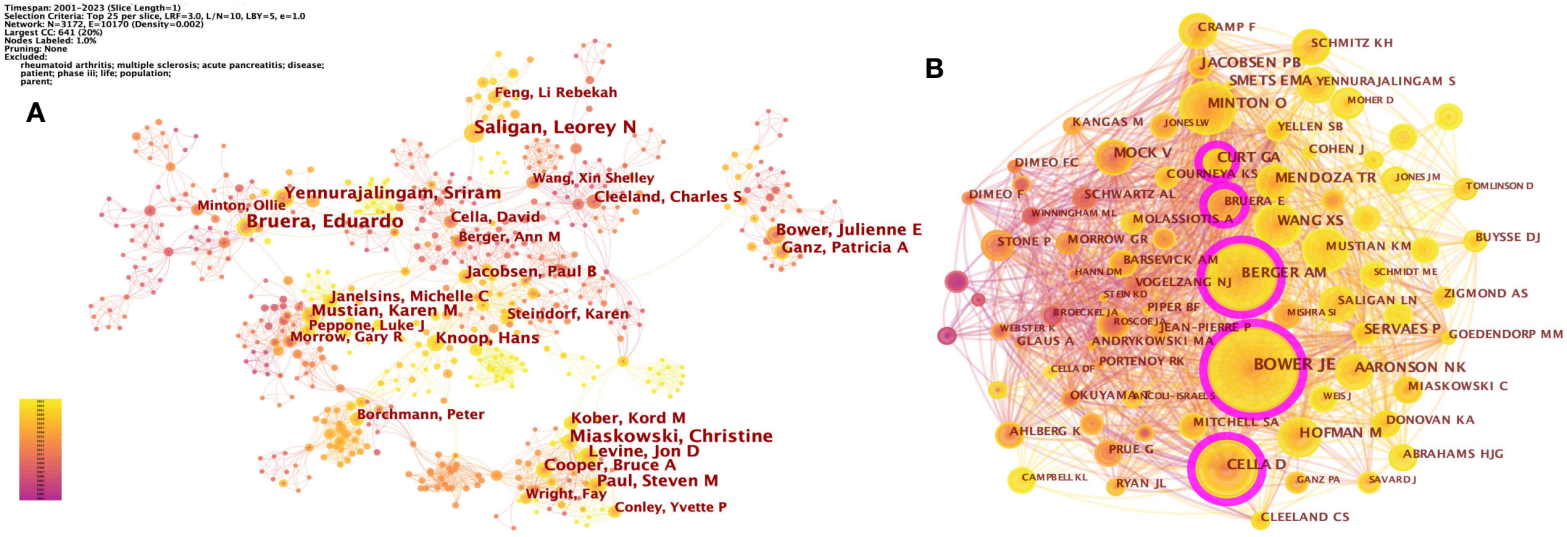
**(A)** The network of co-authorship. **(B)** The network of co-cited authors. Each node and the size of it represent an author/co-cited author and his number of studies/co-citations. Each line represents author collaboration. The different colors indicate the different years of publications. The purple nodes represent that the centrality of co-cited authors is > 0.10.

A total of 10,649 authors published studies about CRF according to CiteSpace. Eduardo Bruera published the most articles (n = 35), followed by Christine Miaskowski (n = 30), Leorey N Saligan (n = 29), Sriram Yennurajalingam (n = 25), and Julienne E Bower (n = 23) (**Table 1**). However, the density of author collaboration was relatively low, reflecting that scholars need to enhance further cooperation in this field. The network map outlines the potential collaboration among authors, including 10,170 connections (**Figure 3A**). Some authors have exhibited close collaborative relationships, such as Julienne E Bower and Patricia A Ganz. The degree of centrality can help us identify hubs in the map, and Paul B Jacobsen has the highest centrality (= 0.04), showing a lack of close cooperation.

**Table 1:** The top 10 authors and co-cited authors in CRF research.

Rank	Author	Count (%)	Centrality	Co-cited author	Co-citation (%)	Centrality
1	Eduardo Bruera	35 (0.24%)	0.01	Julienne E Bower	897 (3.20%)	0.16
2	Christine Miaskowski	30 (0.20%)	0	Ann M Berger	582 (2.07%)	0.13
3	Leorey N Saligan	2 (0.20%)	0.01	David Cella	520 (1.85%)	0.13
4	Sriram Yennurajalingam	25 (0.17%)	0	Ollie Minton	360 (1.39%)	0.03
5	Julienne E Bower	23 (0.16%)	0	G A Curt	355 (1.28%)	0.11
6	Karen M Mustian	22 (0.15%)	0.01	Victoria Mock	312 (1.27%)	0.04
7	Steven M Paul	21 (0.14%)	0	Tito R Mendoza	300 (1.11%)	0.05
8	Jon D Levine	21 (0.14%)	0	Paul B Jacobsen	281 (1.07%)	0.08
9	Patricia A Ganz	20 (0.14%)	0	Wang XS	277 (1.00%)	0.04
10	Hans Knoop	20 (0.14%)	0.03	Patrick Stone	275 (0.99%)	0.06

A co-citation relationship was established among the authors when they were cited simultaneously in one or more articles. The core authors can be shown intuitively through the network map (**Figure 3B**). CiteSpace identified 1,007 co-cited authors, with 25 of them being co-cited over 200 times and 66 being co-cited no less than 100 times. The most co-cited authors were Julienne E Bower (897 co-citations), Ann M Berger (582 co-citations), David Cella (520 co-citations), Ollie Minton (360 co-citations), and G A Curt (355 co-citations) (**Table 1**).

### 3.3 Analysis of co-institution and co-country

**Figure 4:**
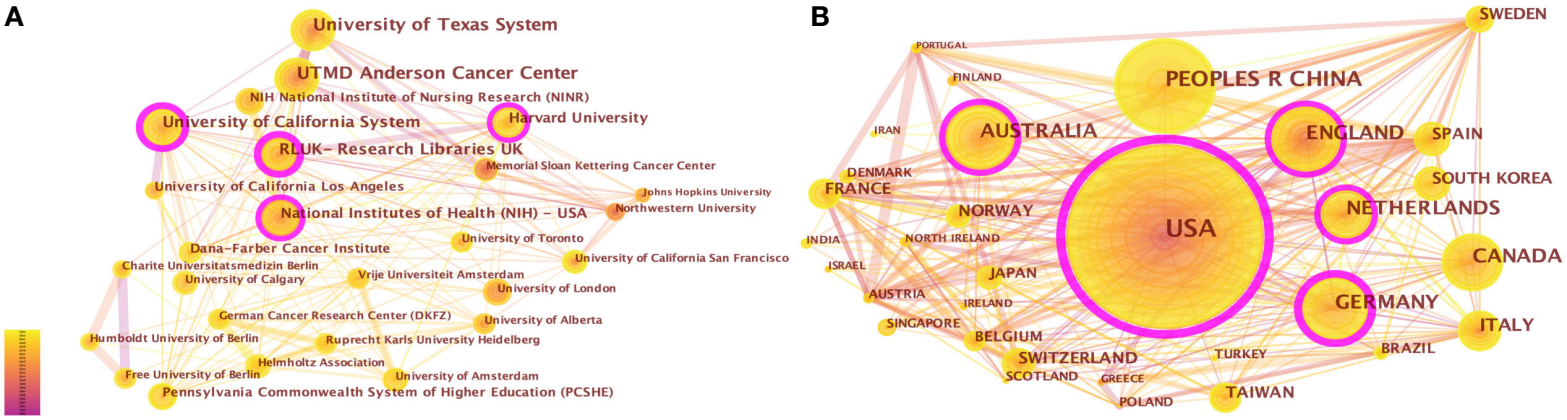
**(A)** The network of co-institutions. **(B)** The network of co-countries. Each node and the size of it represent an institution/country and its number of publications. The thickness of each line represents the strength of collaboration. The different colors indicate the different years of publications. The purple nodes represent that the centrality of co-cited authors is > 0.10.

From 2001 to the present, 1,041 institutions from 70 countries had published studies about CRF. The top 5 productive institutions were the University of Texas System (178, 2.77%), UTMD Anderson Cancer Center (159, 2.48%), University of California System (141, 2.19%), Research Libraries UK (131, 2.04%), and Harvard University (84, 1.31%), four of which were from the USA (**Table 2**). Research Libraries UK exhibited the highest centrality (= 0.36). As can be seen from the pruning network map of co-institution (**Figure 4A**), the collaboration among some institutions is relatively close, such as Anderson Cancer Center and University of Texas System, as well as Research Libraries UK and Harvard University.

**Table 2:** The top 10 institutions and countries in CRF research.

Rank	Institution	N (%)	Centrality	Country	N (%)	Centrality
1	University of Texas System	178 (2.77%)	0.07	USA	1065 (31.26%)	0.50
2	UTMD Anderson Cancer Center	159 (2.48%)	0.05	China	314 (9.22%)	0.05
3	University of California System	141 (2.19%)	0.21	Germany	206 (6.05%)	0.25
4	Research Libraries UK	131 (2.04%)	0.36	England	193 (5.66%)	0.20
5	Harvard University	84 (1.31%)	0.18	Canada	186 (5.46%)	0.05
6	National Institutes of Health-USA	82 (1.28%)	0.11	Australia	179 (5.25%)	0.12
7	Pennsylvania Commonwealth System of Higher Education	59 (0.92%)	0.05	Netherlands	151 (4.43%)	0.12
8	University of California Los Angeles	57 (0.89%)	0.01	Italy	95 (2.79%)	0.06
9	University of Toronto	56 (0.87%)	0.09	Spain	82 (2.41%)	0.04
10	University of London	55 (0.86%)	0.01	France	73 (2.14%)	0.03

**Figure 5:**
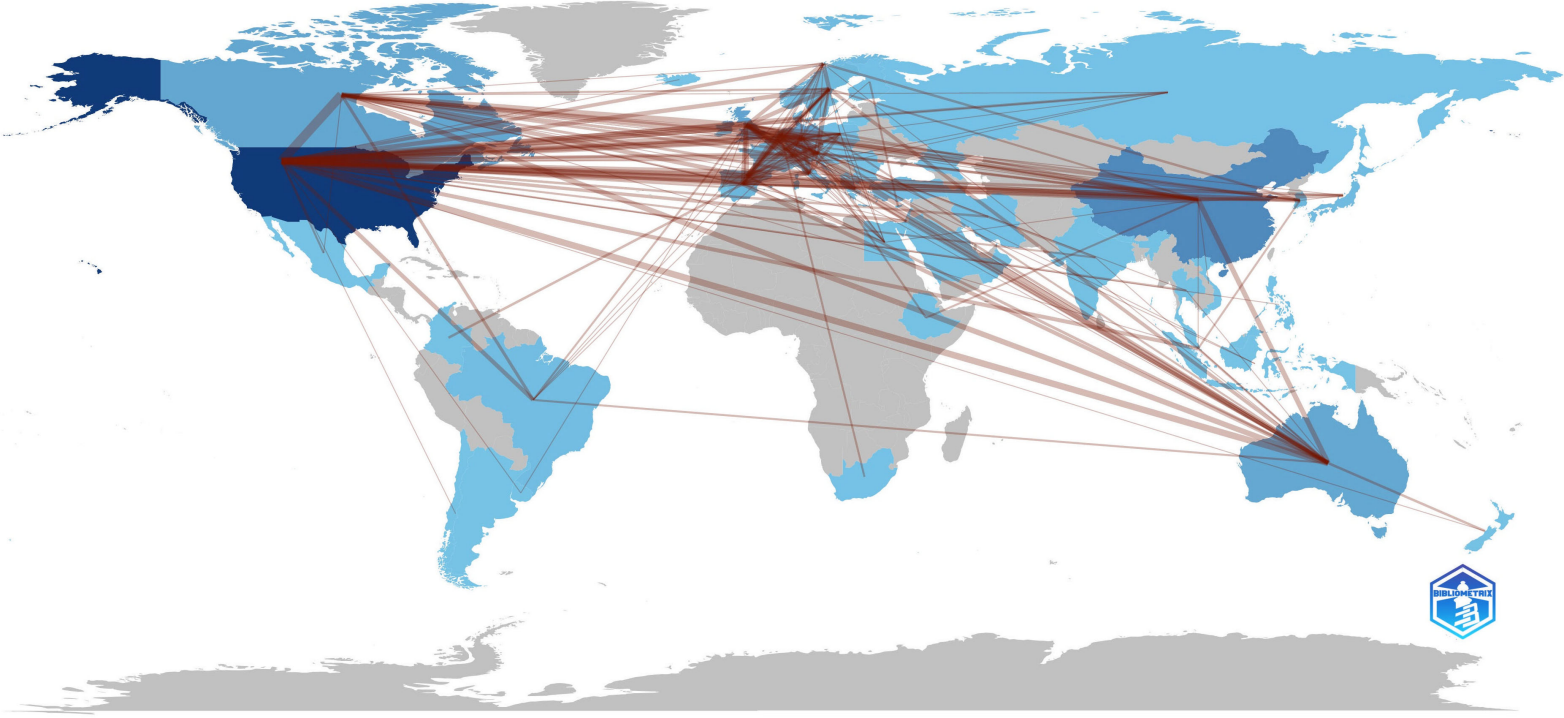
The geographical distribution of global publications.

The United States (1065, 31.26%) had the largest number of publications on CRF, followed by China (314, 9.22%), Germany (206, 6.05%), England (193, 5.66%), and Canada (186, 5.46%) (**Supplementary Table 2**). 70 nodes and 471 connections made up the network map of countries co-occurrence with a density of 0.195 (**Figure 4B**). The USA serves as a pivotal intermediary in the CRF area, exemplifying substantial contributions to the CRF area and assuming the central roles. **Figure 5** visually represents the global distribution of publications, highlighting the cooperation among countries.

### 3.4 Analysis of journals and co-cited journals

650 journals were identified through WoSCC. Supportive Care in Cancer published the most studies (212, 8.26%), followed by Journal of Pain and Symptom Management (116, 4.52%), Cancer (71, 2.77%), Cancer Nursing (62, 2.42%), and Integrative cancer therapies (58, 2.26%) (**Table 3**). Of the top 10 journals publishing the most articles in the CRF field, Cancer had the highest impact factor (IF) of 6.16, while no journals with an IF > 10 were observed.

**Table 3:** The top 10 journals in CRF research.

Rank	Journal	N (%)	IF (2022)	Quartile (2022)
1	Supportive Care in Cancer	212 (8.26%)	3.13	Q2
2	Journal of Pain and Symptom Management	116 (4.52%)	4.70	Q1
3	Cancer	71 (2.77%)	6.16	Q1
4	Cancer Nursing	62 (2.42%)	2.59	Q1
5	Integrative cancer therapies	58 (2.26%)	2.95	Q2
6	Psycho-oncology	58 (2.26%)	3.55	Q2
7	BMC Cancer	44 (1.72%)	3.77	Q2
8	Cancers	41 (1.59%)	5.21	Q2
9	European Journal of Cancer Care	39 (1.52%)	2.07	Q2
10	Oncology Nursing Forum	36 (1.40%)	1.86	Q3

**Figure 6:**
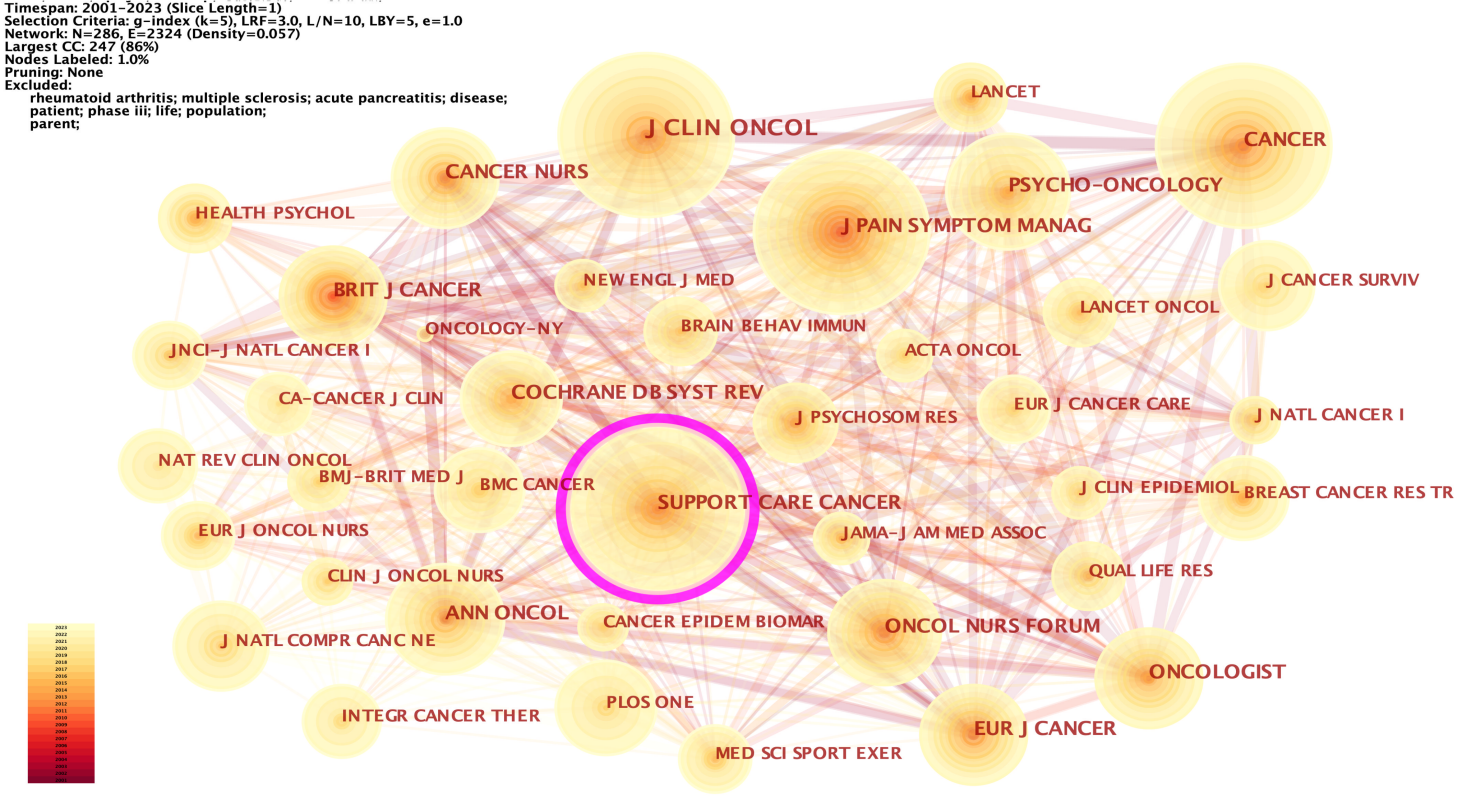
The network of co-cited journals. Each node and the size of it represent a co-cited journal and its number of co-citations. The thickness of each line represents the strength of collaboration. The different colors indicate the different years of publications.

We analyzed the co-cited journals with k = 5 from time slicing of 1 year, and 286 co-cited journals were identified. The co-citations of six journals were more than 1,000, and 20 journals were more than 500. The most co-cited journals were the Journal of Clinical Oncology (1900, 4.41%), Cancer (1806, 4.19%), Supportive Care in Cancer (1674, 3.89%), Journal of Pain and Symptom Management (1562, 3.63%), Annals of Oncology (1080, 2.51%), and Oncology Nursing Forum (1025, 2.38%) (**Supplementary Table 2**). Of these, Annals of Oncology had the highest IF (= 50.45), followed by the Journal of Clinical Oncology (IF = 45.27). 286 nodes and 2,324 connections made up the network map of countries co-occurrence with a density of 0.057 (**Figure 6**). Supportive Care in Cancer had the highest centrality (= 0.11), followed by PLOS ONE (= 0.09), Psycho-oncology (= 0.07), Medicine & Science in Sports & Exercise (= 0.07), and Integrative cancer therapies (= 0.07).

**Figure 7:**
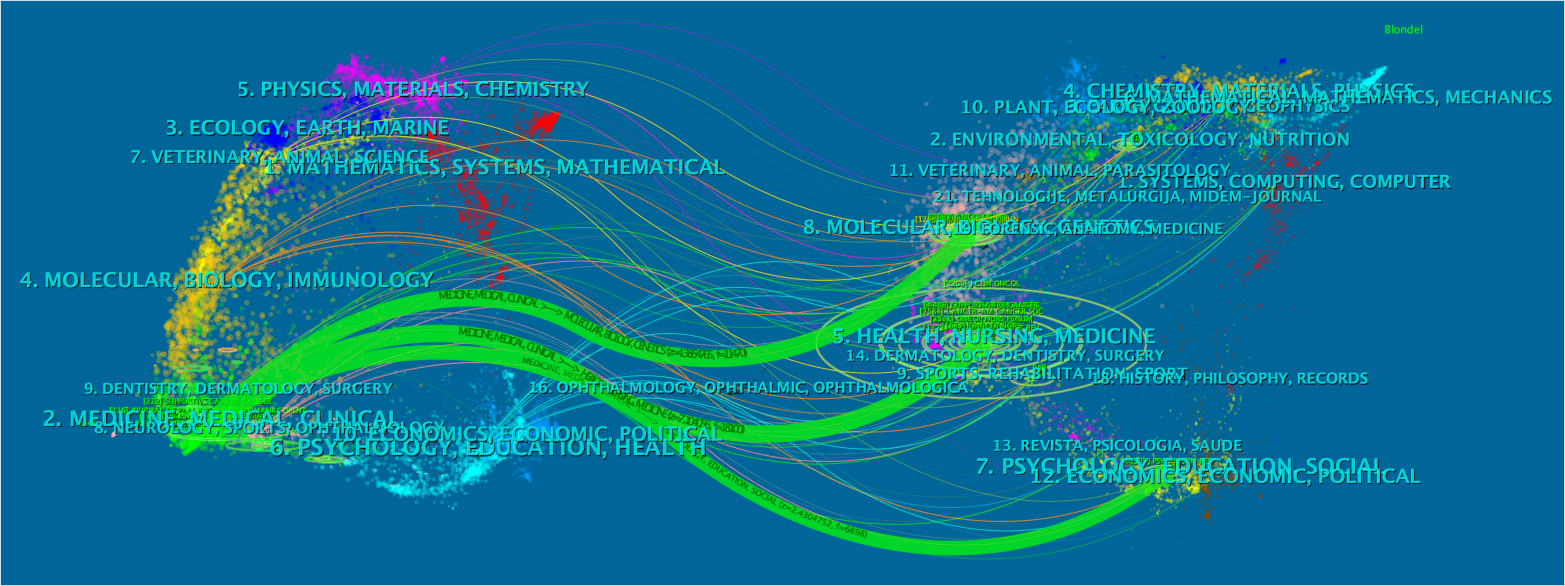
The dual-map overlay of journals. The citing journals were on the left of the map, and the cited ones were on the right. The length of the ellipse represents the number of authors, and the width indicates the count of publications.

The dual-map overlay of the journals revealed the relationship distribution of subject categories among the journals (**Figure 7**). Three predominant citation paths colored with green were identified, which means that the studies published in the Medicine/Medical/Clinical journals were mainly cited by those published in the Molecular/Biology/Genetics (z = 4.39, f = 11,470), Health/Nursing/Medicine (z = 7.10, f = 18,100), and Psychology/Education/Social journals (z = 2.43, f = 6,698).

### 3.5 Analysis of co-cited reference

**Figure 8:**
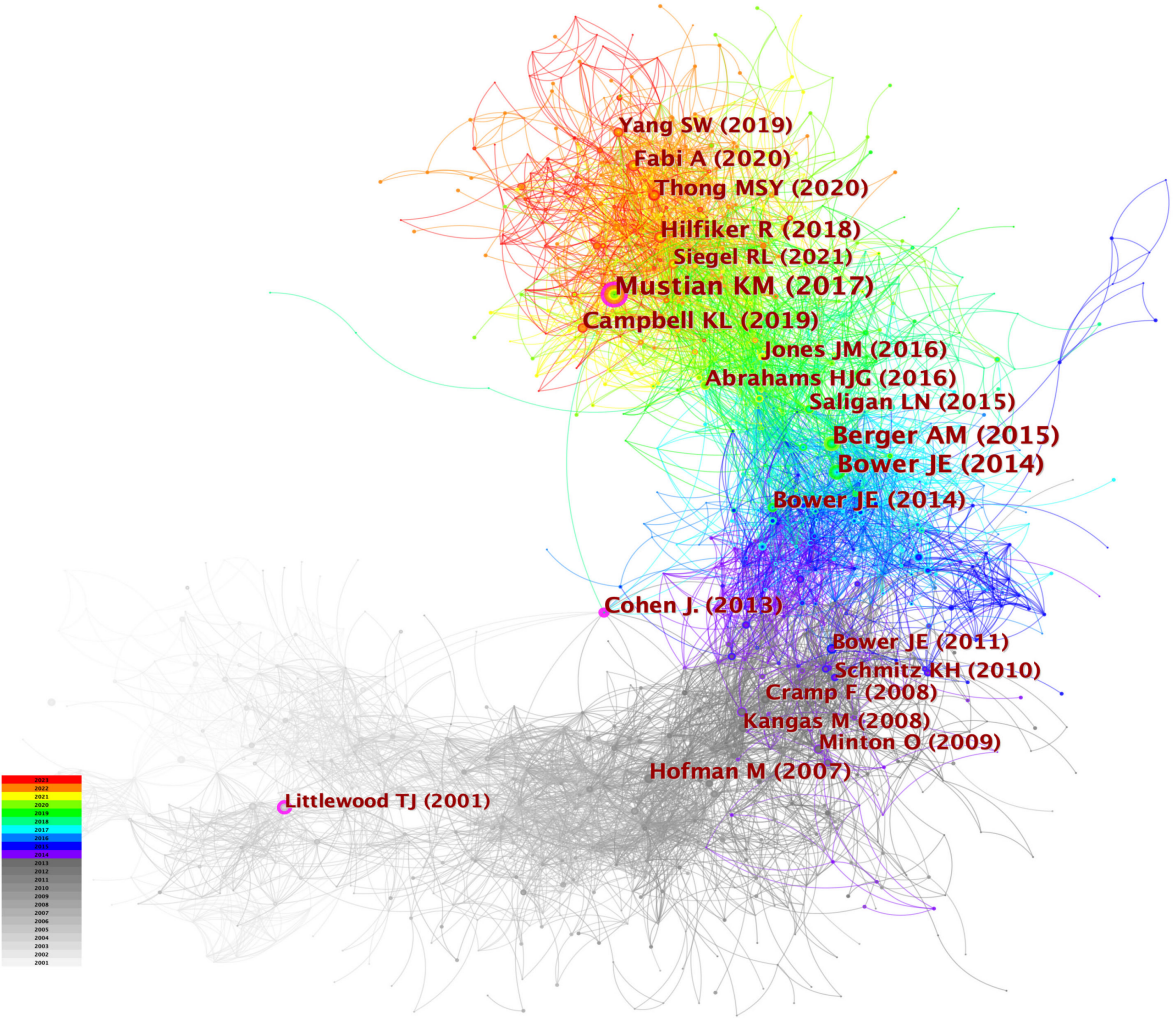
The network of co-cited references. Each node and the size of it represent a co-cited reference and its number of co-citations. The different colors indicate the different years of publications.

When the documents were cited simultaneously, a co-citation relationship was established among them. We analyzed the most co-cited references (k = 25) with a time slicing of 1 year. The core references can be displayed visually through the network map (**Figure 8**), containing 6,980 links with a density of 0.0076. Of 1,360 co-cited references identified through CiteSpace, 13 were co-cited no less than 50 times, and 117 were co-cited no less than 20 times. **Table 4** displays the top 10 co-cited references. “Comparison of Pharmaceutical, Psychological, and Exercise Treatments for Cancer-Related Fatigue: A Meta-analysis”, authored by Mustian KM et al. and published in JAMA Oncology was the most co-cited document in the CRF area, which indicated that exercise and psychological treatments were more effective than pharmaceutical interventions for CRF through meta-analyses (9). Berger AM et al. provided recommendations for screening and treatments of CRF (10). Bower JE et al. summarized the possible mechanisms, risk factors, and treatments for CRF (4). Campbell KL et al. provided a consensus statement about exercise recommendations for cancer survivors (11). Hilfiker R et al. (12) evaluated the effects of non-pharmaceutical interventions on CRF. Overall, 6 of the top 10 most co-cited documents were reviews, and 4 were guidelines.

**Table 4:** The top 10 co-cited references in CRF research.

Rank	Author	Cited reference	Co-citation (%)	Centrality	Journal	IF (2021)
1	Mustian KM (9)	Comparison of Pharmaceutical, Psychological, and Exercise Treatments for Cancer-Related Fatigue: A Meta-analysis	133 (1.26%)	0.16	JAMA Oncology	28.37
2	Berger AM (10)	Cancer-Related Fatigue, Version 2.2015	103 (0.98%)	0.01	Journal of the National Comprehensive Cancer Network	13.44
3	Bower JE (4)	Cancer-related fatigue–mechanisms, risk factors, and treatments	99 (0.94%)	0	Nature Reviews Clinical Oncology	78.77
4	Campbell KL (11)	Exercise Guidelines for Cancer Survivors: Consensus Statement from International Multidisciplinary Roundtable	62 (0.59%)	0.01	Medicine & Science in Sports & Exercise	4.15
5	Hilfiker R (12)	Exercise and other non-pharmaceutical interventions for cancer-related fatigue in patients during or after cancer treatment: a systematic review incorporating an indirect-comparisons meta-analysis	61 (0.58%)	0.05	British Journal of Sports Medicine	18.42
6	Bower JE (13)	Screening, assessment, and management of fatigue in adult survivors of cancer: an American Society of Clinical oncology clinical practice guideline adaptation	61 (0.58%)	0.06	Journal of Clinical Oncology	45.27
7	Abrahams HJG (14)	Risk factors, prevalence, and course of severe fatigue after breast cancer treatment: a meta-analysis involving 12 327 breast cancer survivors	54 (0.51%)	0.09	Annals of Oncology	50.45
8	Fabi A (15)	Cancer-related fatigue: ESMO Clinical Practice Guidelines for diagnosis and treatment	53 (0.50%)	0.01	Annals of Oncology	50.45
9	Hofman M (16)	Cancer-related fatigue: the scale of the problem	53 (0.50%)	0	Oncologist	5.76
10	Saligan LN (17)	The biology of cancer-related fatigue: a review of the literature	52 (0.49%)	0.04	Supportive Care in Cancer	3.13

**Figure 9:**
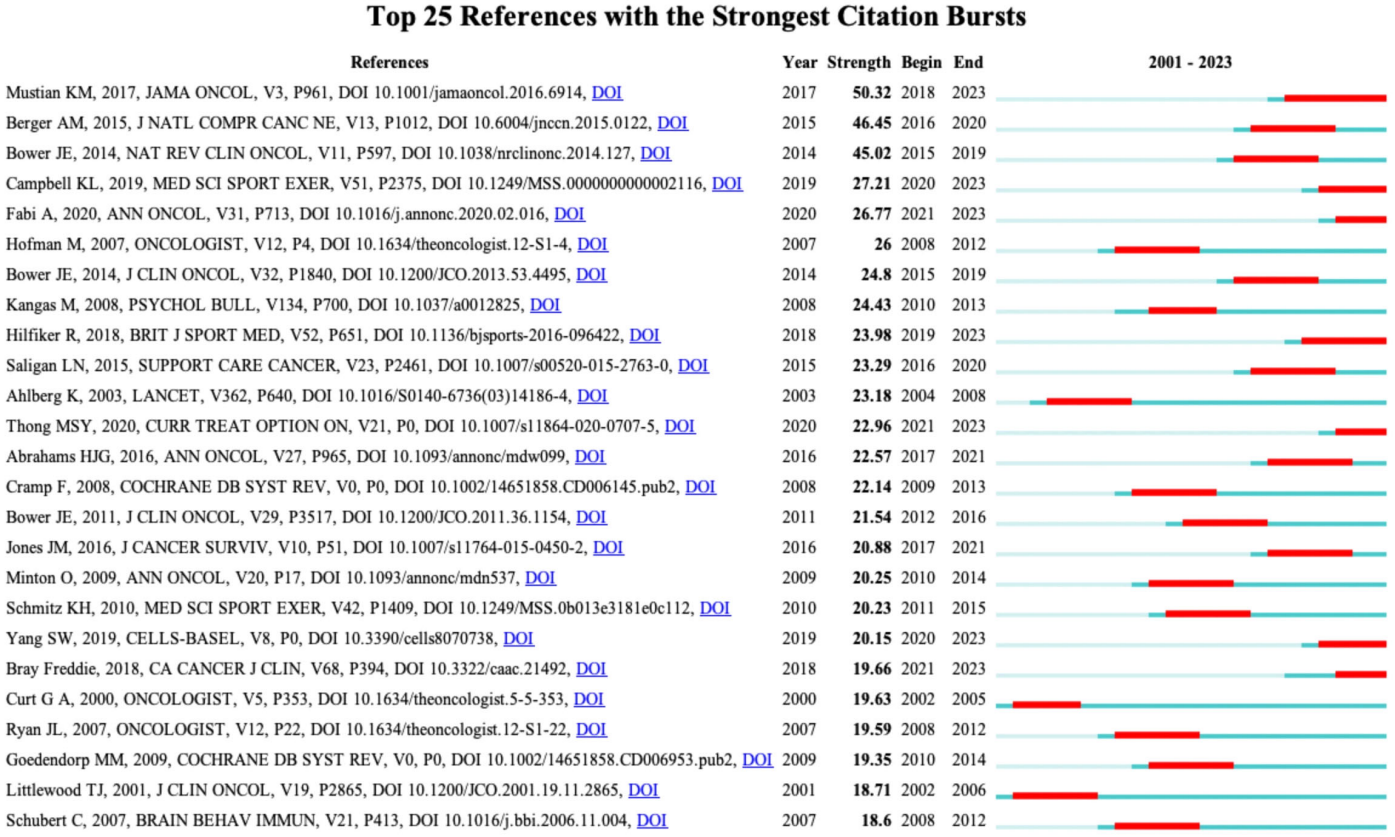
The top 25 references with strongest citation bursts. Red lines represent the duration of citation bursts. The figure was arranged in descending order based on the strength of citation bursts.

**Figure 9** displays the top 25 documents with the strongest citation bursts, showing growing interest in the emerging field. The earliest beginning year of citation burst of reference was from 2002 to 2005 (strength = 19.63), entitled “Impact of Cancer-Related Fatigue on the Lives of Patients: New Findings From the Fatigue Coalition” (18) by Curt GA et al. and published in Oncologist. “Comparison of Pharmaceutical, Psychological, and Exercise Treatments for Cancer-Related Fatigue: A Meta-analysis” (9) published by Mustian KM et al. had the strongest strength of 50.32, which was also the most co-cited reference.

### 3.6 Analysis of keyword

**Figure 10:**
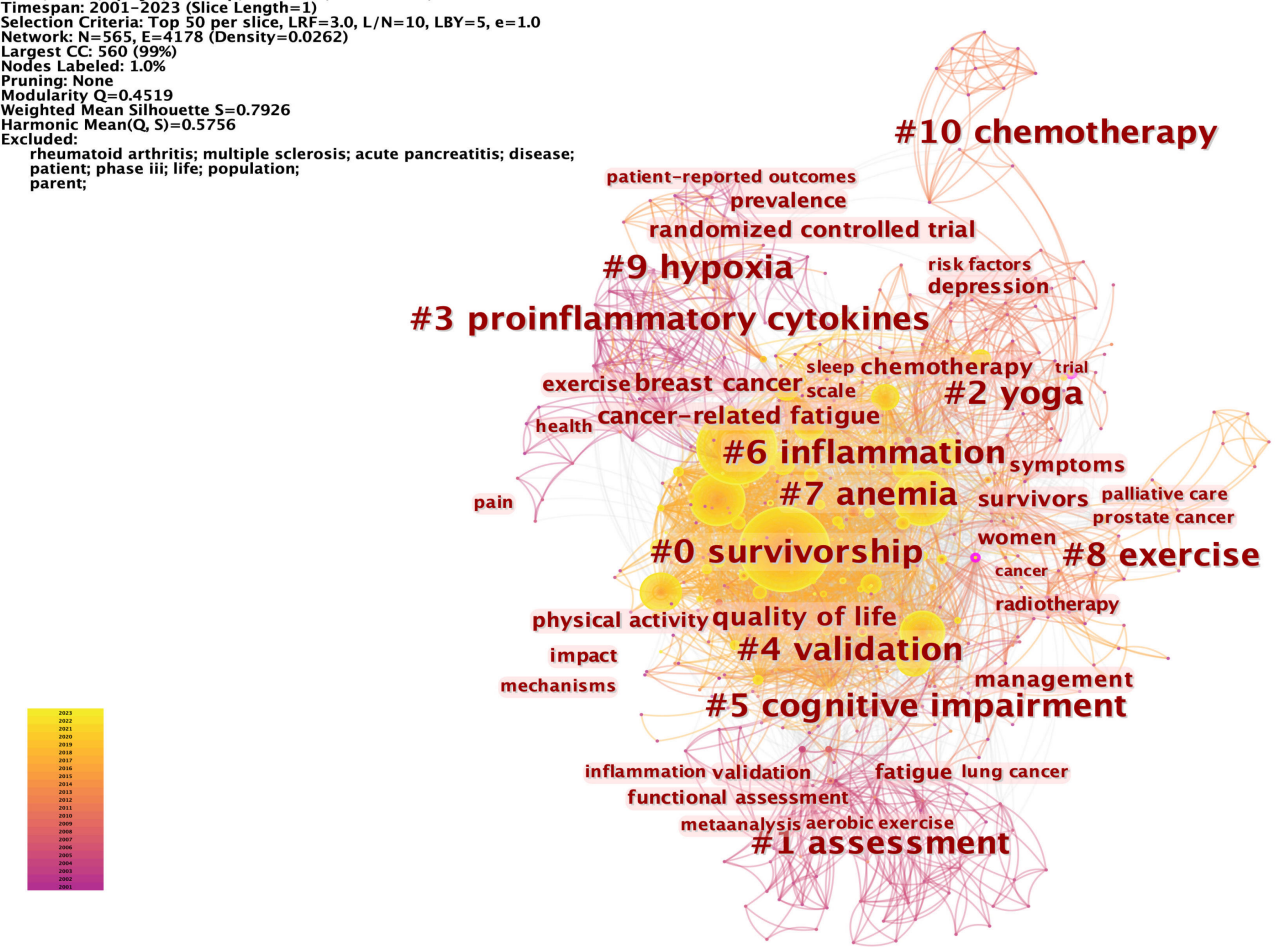
The network of the top 50 keywords co-occurrence and the cluster analysis. Each node and the size of it represent a co-cited reference and its number of co-citations. The different colors indicate the different years of publications. The phrases in # represent the clusters of keywords.

Keywords represent a high generalization of the central theme of an article. Analyzing keywords can help to identify the research hot spots in the CRF field over a period of time. We analyzed the top 50 highest-frequency keywords in each time slice. 565 nodes and 4,178 connections made up the network map of keywords co-occurrence with a density of 0.0262 (**Figure 10**). Of 565 keywords identified through CiteSpace, 31 appeared over 100 times, and 111 appeared no less than 10 times. **Supplementary Table 3** presents the top 20 keywords in CRF research. The main contents of the CRF field were identified as “Quality of life” (1315), “breast cancer” (841), “survivors” (614), “management” (564), “chemotherapy” (471), “physical activity” (370), and “depression” (355). 10 clusters were revealed in the cluster analysis of keywords through CiteSpace (**Figure 10**), including survivorship, assessment, yoga, proinflammatory cytokines, validation, cognitive impairment, inflammation, anemia, exercise, hypoxia, and chemotherapy.

**Figure 11:**
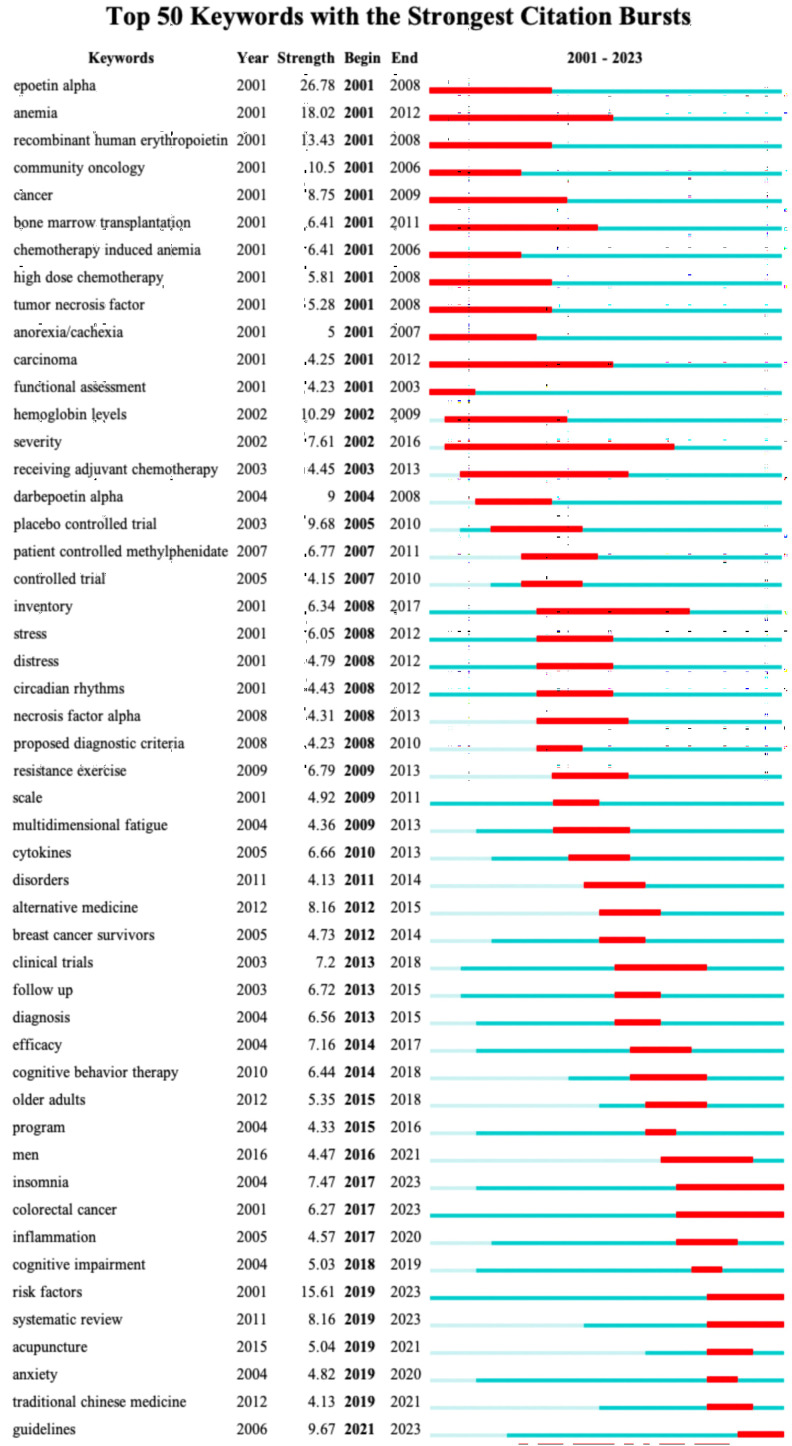
The top 50 keywords with strongest citation bursts. Red lines represent the duration of citation bursts. The figure was arranged in ascending order based on the beginning year of burst.

**Figure 11** displays the top 50 keywords with the strongest citation bursts, which elucidates the evolving patterns of focal points in the CRF area. Notably, the keyword “epoetin alpha” was the strongest burst (strength = 26.78) and also has the earliest year of citation bursts (2001). The keywords “risk factors,” “systematic review,” “acupuncture,” “anxiety,” and “traditional Chinese medicine” appeared in 2019, while “guidelines” occurred in 2021, which were the most recent citation bursts. Moreover, “insomnia,” “colorectal cancer,” “risk factors,” “systematic review,” and “guidelines” have continued to be of ongoing interest until the year 2023.

## 4 Discussion

### 4.1 Summary of findings

This study conducted bibliometric and visualization analyses using CiteSpace to examine 2,566 scientific articles published between 2001 and 2023, providing comprehensive knowledge network maps of CRF. The findings indicate that CRF has garnered extensive attention since 2001, with a notable surge in interest observed in 2019, showing sustained attention towards CRF over the coming years. The most prolific author is Eduardo Bruera from the UTMD Anderson Cancer Center, and the most co-cited author is Julienne E Bower from the University of California. Additionally, the University of Texas System is the most productive institution. Nine countries (the USA, Germany, England, Canada, Netherlands, Italy, France, Japan, and Greece) have embarked on comprehensive research on CRF since 2001. Here in this article the United States and China made the most contributions to this field. However, the low centrality of China highlights the need for Chinese scholars to enhance international association and collaboration.

Supportive Care in Cancer published the most studies on CRF, while Journal of Clinical Oncology has the highest number of co-citations. Most-cited references generally address the possible mechanisms and treatments of CRF. The top ten keywords with the highest frequency of occurrence are quality of life, cancer-related fatigue, breast cancer, survivors, management, chemotherapy, randomized controlled trial, fatigue, physical activity, and depression. Our analysis of keywords with the strongest citation bursts have revealed that “yoga,” “inflammation,” “hypoxia,” “anemia,” “exercise,” “acupuncture,” “anxiety,” “traditional Chinese medicine,” and “insomnia” might be emerging hotspots in the CRF area.

### 4.2 Research hotspots on CRF

We summarized the hotspots in CRF research based on the results obtained from CiteSpace, primarily encompassing risk factors (anemia, insomnia, depression, and anxiety), treatments (exercise, yoga, acupuncture, and traditional Chinese medicine), and mechanisms (inflammation and hypoxia) of CRF.

#### 4.2.1 Risk factors

Previous reports have indicated that several factors, including anemia, insomnia, anxiety, and depression, are essential for the occurrence and development of fatigue in patients with cancer (19–21).

Patients with cancer often have anemia, especially during or after chemotherapy. Low hemoglobin levels often cause a decrease in blood oxygen levels, leading to the development of some fatigue-related symptoms. These findings have prompted attempts to investigate the correlations between cancer-related anemia and CRF. Some evidence shows that administration of epoetin or darbepoetin to increase hemoglobin level is an effective therapy for reducing CRF (19). However, this treatment may be limited to patients with cancer-related anemia caused by hematological malignancies, such as lymphoma or multiple myeloma, or cancer treatments, such as chemotherapy (22, 23). The analysis conducted in the present study revealed that the keywords “epoetin alpha” and “anemia” have the earliest year of citation bursts (2001); however, this surge only lasted until 2012, indicating that the effect of anemia on CRF has not been a topic of interest in recent years.

Approximately 25-59% of patients with cancer experience insomnia. Insomnia is a prevalent type of sleep disorder characterized by poor sleep quality and difficulties with sleep initiation and maintenance (24). The National Comprehensive Cancer Network (NCCN) has identified that insomnia has a critical influence on CRF (1). A meta-analysis of 144,813 patients with cancer indicated that insomnia has a significant effect on CRF (OR = 2.83), and that improved insomnia is associated with decreased levels of CRF (2). Several possible mechanisms, such as alterations in the circadian rhythm, dysregulation of the hypothalamic-pituitary-adrenal (HPA) axis, inflammatory and immune responses, and abnormal neurotransmitter levels, may be implicated in the correlation between insomnia and CRF (25).

Anxiety and depression are common mental health problems that affect approximately 10-20% of patients with cancer. The correlation between CRF and anxiety/depression is undeniable. However, the association between CRF and depression seems to receive more attention than that between CRF and anxiety (26–28). A systematic review of 59 studies showed that the correlation between anxiety (0.46) and CRF is weaker than that between depression and CRF (0.56) (29). However, several studies have indicated that anxiety may have a more pronounced effect on CRF than depression in the early stages of cancer, which may be attributed to the predominance of anxiety as the primary emotional response in this stage (30). It is believed that anxiety/depression may contribute to loss of appetite, decreased engagement in physical activities, muscle tension, or disrupted sleep, all of which are associated with the occurrence or increased levels of CRF. The precise mechanisms underlying the relationship between anxiety/depression and CRF are multifaceted and not completely understood; however, they may involve dysregulation of the HPA axis, alterations in neurotransmitter levels, and inflammatory responses (31).

In addition to anxiety and depression, there are many psychosocial risk factors of CRF, such as childhood stress, loneliness, and “catastrophize” of cancer. Conversely, CRF can also impact social, cognitive, and emotional functioning (4). The effects of CRF on social, cognitive, and emotional functioning is an integral component in various CRF scales. Moreover, recent research indicates that CRF, along with multiple cancer-related symptoms such as depression, anxiety, insomnia, poor concentration, and other cognitive impairments, is increasingly being reported as symptom clusters (32), which indicates that CRF and these symptoms may share similar mechanisms and interactions. The amelioration of one symptom is likely to improve others. Studying of associated risk factors contributes to early assessment, diagnosis, and management of CRF. Improving social, cognitive, and emotional functioning status of patients aids in reducing CRF, and avoiding the vicious cycle of “fatigue-impairment in social/cognitive/emotional functioning-fatigue exacerbation.”

#### 4.2.2 Treatments

Over the past two decades, multiple randomized controlled trials have been conducted with CRF as a primary or secondary outcome measure. These studies have demonstrated diverse effective management strategies for CRF (4), especially non-pharmacological interventions such as exercise, yoga, and acupuncture.

Existing evidence suggests that exercise is the most effective therapeutic option for preventing and improving CRF and can be performed by patients with cancer of any stage (8, 15). The exercise guidelines (11) for cancer survivors recommend that engaging in moderately intense aerobic exercise (three times per week) for at least 12 weeks can significantly reduce CRF. A meta-analysis (12) of 245 studies revealed that among all types of exercise, relaxation exercises may be preferable for patients with CRF during cancer treatment. However, another study (33) concluded that a combination of aerobic and resistance exercises yields the most improvements in CRF, either during or after cancer treatment. In addition, it has been reported that aerobic exercise for improving CRF is more beneficial for patients with solid tumors than for those with hematological malignancies (34). It is important to consider variations in the type, duration, and intensity of exercise for different populations, such as patients with cancer who have bone metastases, anemia, or fever (1). Further research is warranted to provide evidence of the benefits of exercise in different populations of patients with CRF.

Yoga, a type of mind-body exercise, is widely performed to relieve cancer-related symptoms, particularly fatigue. Current guidelines recommend performing yoga to reduce CRF during and after cancer treatment (1, 15). A Cochrane review (35) of 24 studies provided moderate-quality evidence that yoga improves CRF in patients with breast cancer compared with psychosocial/educational interventions. A meta-analysis (36) of 2,183 patients with breast cancer showed that an eight-week yoga regimen significantly mitigated the physical fatigue of the patients but had little effects on cognitive/mental fatigue. Yoga can regulate the dysregulation of the HPA axis, decrease plasma cortisol levels, and increase the release of endogenous dopamine and γ-aminobutyric acid levels (37–39). Some studies have been conducted to investigate the potential mechanisms underlying the improvement effect of yoga on CRF (40, 41), which may involve the modulation of the inflammatory processes, such as regulating the levels of inflammatory cytokines (e.g., IL-1β, IL-10), or the NF-κB pathway. Moreover, yoga may enhance glucocorticoid signal transduction and reduce sympathetic nervous system signaling through β-adrenergic receptors to influence inflammatory processes.

The American Society of Oncology and NCCN guidelines recommend acupuncture, an essential component of traditional Chinese medicine (TCM), for attenuating CRF in patients after cancer treatment (1, 13). A meta-analysis (42) of 1,084 patients with breast cancer revealed that acupuncture can significantly reduce CRF compared with sham acupuncture or usual care. A minimum of six acupuncture sessions (e.g., weekly for 6 weeks, three times per week for 3 weeks), each lasting at least 20 minutes, has significant effects on CRF (43). Two studies (44, 45) demonstrated that acupuncture can inhibit neuroinflammation and gut inflammation by regulating the gut microbiota-gut-brain axis, decrease serum ghrelin, leptin, and insulin levels, and improve the dysfunction of the HPA axis to improve CRF. However, as further studies are still needed to verify the effects of acupuncture on CRF, the European Society for Medical Oncology panel has not yet reached a consensus on the recommendation of acupuncture for reducing CRF.

In addition to acupuncture, Chinese herbal medicines and Chinese medicine compound prescriptions have been shown to reduce CRF. Ginseng, a commonly used Chinese herbal medicine, tonifies the qi, nourishes the blood, and strengthens the spleen. A randomized trial (N07C2) (46) provided evidence to support the effects of American ginseng (Panax quinquefolius) (2000 mg/day) on CRF, and no discernible treatment-related toxicities are reported. Three systematic reviews (47–49) have demonstrated that ginseng, including Asian, red, and American ginseng, can alleviate CRF. However, no clear criteria regarding the duration and dose of ginseng that should be administered to alleviate CRF have been established. Moreover, the effects of ginseng in reducing CRF may be influenced by various factors such as the type of ginseng, method of preparation, and ginsenoside content. In addition, huangqi (Radix Astragali), baizhu (Atractylodes macrocephala Koidz), and shanyao (Chinese yam) may regulate the levels of cytokines such as tumor necrosis factor-alpha (TNF-α) and interleukin-6 (IL-6), and inhibit oxidative stress to reduce CRF (50, 51).

Shengqi Fuzheng injection, which mainly consists of dangshen (Radix Codonopsis) and huangqi, improves mitochondrial dysfunction through the PI3K/Akt and AMPK signaling pathways, inhibits the levels of pro-inflammatory cytokines, and enhances immune function by downregulating multiple targets (PD-L1, TIM3, and FOXP3) to alleviate CRF (52, 53). Moreover, the effects of the Buzhong Yiqi decoction, Jianpi Shengsui Gao, and Shiquan Dabu decoction have been validated in animal studies and clinical trials (54–56). However, the evidence supporting these findings is of low quality; as such, TCM is not recommended for the treatment of CRF in any guideline. Therefore, further research on the effects of TCM on CRF is required.

#### 4.2.3 Mechanisms

##### 4.2.3.1 Inflammation

The analysis conducted in this study indicated that inflammation is a hot topic in studies on the mechanisms of CRF (4). There are large variations in the symptoms experienced by both animals and humans after infections (57). These symptoms, including fatigue, significant changes in sleep patterns, and loss of appetite, are largely related to the effects of pro-inflammatory cytokines on the central nervous system. Persistent inflammation also plays an important role in CRF (58–60), primarily because of the activation of a network of pro-inflammatory cytokines by anti-tumor therapies. A study (61) demonstrated that 65% of patients with acute myelogenous leukemia/myelodysplastic syndrome experience CRF before treatment, which is associated with IL-1RA, IL-6, and TNF-α levels. Chemotherapy and radiotherapy are commonly administered to treat cancer. Chemotherapy with platinum- or anthracycline-based agents may increase IL-8 and IL-6 levels, thereby leading to CRF (62, 63). Increased serum levels of C-reactive protein (CRP) and IL-1RA are associated with increased CRF in patients undergoing radiotherapy (64). This correlation has also been observed in patients with advanced cancer (65, 66). Most breast cancer survivors with fatigue show upregulated expression of genes encoding pro-inflammatory cytokines compared with non-fatigued patients. In addition, cancer survivors with fatigue show increased expression of transcripts with response elements for NF-κB (67).

Other factors, such as psychological distress and insufficient sleep, can also affect inflammatory responses. Stress can activate peripheral inflammatory cytokines and related signaling pathways, such as the NF-κB signaling pathway, which are mediated by the activation of the sympathetic nervous system and the release of catecholamines (68–70). A meta-analysis (71) showed that acute stress may lead to marked increase in circulating IL-1β, IL-6, IL-10, and TNF-α levels. In addition, insufficient sleep can lead to elevated CRP, IL-6, and TNF-α levels, which may be associated with reduced cortisol secretion and disrupted circadian rhythm (72–74). Peripheral pro-inflammatory cytokines can access the brain through various pathways, activating microglia and astrocytes and contributing to neuroinflammation, which is a significant reason for the depression-like behaviors experienced by patients with CRF. Multiple inflammatory biomarkers, including CRP, IL-1RA, IL-6, sTNFR2, TNF-α, have been shown to be strongly associated with CRF. In addition, some of these biomarkers have been assessed as independent risk factors for CRF (17, 75–78). Moreover, some studies have confirmed that improvements in the levels of certain inflammatory markers can ameliorate CRF (40, 79–81).

Although the specific mechanism by which inflammation affects CRF remains elusive, it has been established that inflammation is intricately linked to neurotransmitter metabolism, HPA axis regulation, and skeletal muscle and mitochondrial dysfunction. Pro-inflammatory cytokines, such as IL-1β, IL-6, IFN-α, IFN-γ, and TNF-α, can activate the indoleamine 2,3-dioxygenase (IDO) pathway, which increases the degradation of tryptophan into kynurenine through the kynurenine (KYN) pathway, leading to reduced serotonin levels (82). Serotonin, an essential neurotransmitter, influences depression and sleep; however, its effect on CRF remains controversial (83, 84). The activation of the KYN pathway can also affect the functions of dopamine and norepinephrine. Kynurenic acid, generated from the degradation of kynurenine, can inhibit glutamatergic function, which may suppress the release of dopamine and result in CRF (82, 85). Methylphenidate, one of the few pharmacological therapies recommended for CRF in current clinical guidelines, acts by elevating dopamine levels (86).

Pro-inflammatory cytokines can influence the function of the HPA axis. The HPA axis regulates the generation of cytokines to exert anti-inflammatory effects through negative feedback control mechanisms (4). Cortisol, a glucocorticoid released by the HPA axis in response to factors such as inflammation, exhibits a clear circadian rhythm in healthy individuals. The diurnal cortisol rhythm appears flatter in cancer survivors with fatigue, indicating that patients with CRF experience disruptions in the circadian cortisol rhythm (87, 88). Notably, the flattened cortisol secretion slope in cancer survivors with fatigue is attributed to elevated cortisol levels in the afternoon and evening. Furthermore, a study demonstrated that breast cancer survivors with fatigue exhibit diminished expression of glucocorticoid receptor anti-inflammatory transcription factors, indicating the presence of functional resistance to glucocorticoids, which may lead to the persistence of inflammatory responses (89).

Inflammation mediates mitochondrial dysfunction through various signaling pathways. For instance, IL-6 can activate STAT3 to disrupt homeostasis in skeletal muscles, whereas TNF-α can promote protein degradation in skeletal muscles by activating the NF-κB signaling pathway (90, 91). Studies have shown that inflammation increases the mitochondrial localization of NLRP3 and enhances the production of mitochondrial reactive oxygen species (ROS) (92). Dysfunctional mitochondria release excessive ROS and diminish adenosine triphosphate generation (93). Long-term elevation of the levels of ROS induces DNA damage, protein oxidation, and cellular apoptosis. Additionally, ROS can affect skeletal muscle function by interfering with the Akt/mTOR signaling pathway and augmenting protein hydrolysis (94). Mitochondrial and skeletal muscle dysfunction are important factors that influence CRF.

##### 4.2.3.2 Hypoxia

Metabolic activities in immune cells change after inflammatory stimulation, such as increased oxygen consumption by activated neutrophils and elevation of glycolytic rates by activated macrophages and lymphocytes, leading to hypoxia (95). Studies show that mitochondrial dysfunction can exacerbate hypoxia within the tumor microenvironment. Under anoxic conditions, the hypoxia-inducible factor (HIF) is activated and serves as the dominant regulator of oxygen homeostasis (96). The HIF-α and HIF-β subunits induce transcription of multiple genes, such as NF-κB and toll-like receptors, promote the release of chemokines and cytokines, and stimulate the activation and recruitment of phagocytes and leukocytes to regulate the function of immune cells (97, 98). HIF-1 also has the capacity to upregulate the expression of NADPH oxidase, augmenting the generation of ROS within the cytoplasm to promote oxidative stress (99, 100). In turn, ROS can enhance the stability of HIF-1 and facilitate the activation of both HIF-1 and NF-κB. Jianpi Shengsui Gao, a kind of TCM, enhances antioxidant stress via the HIF-1 pathway to mitigate CRF (55).

Hypoxia-induced oxidative damage alters mitochondrial morphology, metabolism, and respiration (101). Mitochondrial autophagy is an adaptive metabolic response and a crucial mechanism for eliminating oxidatively damaged mitochondria, which contributes to the maintenance of energy metabolism. HIF induces mitochondrial autophagy by modulating the expression of BNIP3/Beclin-1 (102, 103). PHD2 is a key enzyme involved in the regulation of HIF-1α stability. A study showed that acteoside from Cistanche tubulosa can promote mitochondrial autophagy by suppressing PHD2 to reduce CRF (104). Existing evidence indicates that the HIF pathway may serve as a pivotal target in the treatment of chronic inflammation, which also plays a role in immune regulation and antitumor activity (105–108). Although research on the HIF pathway is still in its nascent stage, particularly in the context of CRF, the HIF pathway represents an exciting therapeutic target that warrants further investigation.

Whether CRF originated in the central or peripheral remains controversial. Previous studies examining electroencephalogram (EEG), fatigue-related muscle contractile properties, and electromyogram (EMG) supported the findings of central origin of CRF (109–113). Another study quantifying EEG-EMG coherence indicated significant and robust weakening of corticomuscular signal coupling in CRF patients, which may be caused by central and peripheral neuropathies (114). Although the mechanism of neuroelectric activity of CRF is still unclear, improving neuromuscular transmission may also be an important method for CRF treatment. Certainly, more research is needed. On the other hand, whether CRF is of central or peripheral origin, inflammation may play a central role. Pro-inflammatory cytokines can promote neuroinflammation through multiple pathways, affecting central nervous system function and neurotransmitter metabolism. Inflammation is also related to skeletal muscle mitochondrial function. The joint action of inflammation and skeletal muscle mitochondria can lead to hypoxia and abnormal oxidative stress response, which in turn aggravates inflammation and skeletal muscle mitochondrial dysfunction. This study indicates the importance of inflammation in the CRF field, and hypoxia is a possible research direction in the future. Targeting related pathways may be a new method to reduce CRF.

### 4.3 Emerging areas on CRF

The results of this analysis indicate that recent research on the risk factors for CRF has predominantly been focused on insomnia, anxiety, and depression. Anemia remains a risk factor for CRF, and erythropoietin is recommended for patients with CRF and concomitant anemia (1). The main potential mechanisms by which insomnia, anxiety, and depression cause CRF include inflammation, HPA axis dysregulation, neurotransmitter alterations, and disturbances in circadian rhythms. Analysis of the keywords with the strongest citation bursts highlights inflammation as current hotspot in research on the mechanisms underlying the development of CRF. Inflammation is closely associated with HPA axis dysfunction, alterations in neurotransmitters, disruptions in circadian rhythms, and skeletal muscle and mitochondrial dysfunction, all of which collectively exert a synergistic effect on the onset and progression of CRF. Several studies have indicated that modulating the levels of pro-inflammatory cytokines, such as IL-1RA, IL-6, and TNF-α, to ameliorate systemic and neural inflammation can effectively alleviate CRF (79–81).

Cluster analysis of keywords indicated that hypoxia is a potential mechanism underlying the development of CRF, possibly representing an emerging topic for future studies in the field of CRF research. HIF-related pathways are activated in hypoxic conditions, which can modulate the functions of immune cells, release of inflammatory cytokines, oxidative stress status, and mitochondrial function. Targeting HIF-related pathways has been proven to be a possible therapeutic strategy for various chronic inflammatory and neurodegenerative diseases (115) and may potentially aid the improvement of CRF (55, 104). However, further substantiation through more evidence is warranted to determine whether targeting HIF-related pathways can ameliorate CRF.

The findings of this study highlight the current research hotspots regarding the risk factors, treatments, and mechanisms of CRF, and provide novel insights into future therapeutic directions for CRF. Notably, the results of this study indicated that hypoxia, which is closely associated with inflammation, is one of the less explored mechanisms underlying the development of CRF. Further studies are needed to clarify whether hypoxia-associated pathways can be crucial targets in the treatment of CRF.

Treatment strategies for CRF have been evaluated in previous studies. According to existing guidelines, exercise is the most effective intervention for improving CRF. However, there is no consensus on the optimal type, frequency, and intensity of exercise suitable for various types and stages of cancer. Future in-depth studies on this topic are needed to aid clinicians in designing optimal treatment strategies for patients with CRF. Acupuncture has demonstrated significant efficacy in ameliorating cancer-related symptoms. However, future extensive, large-scale, clinical studies are needed to verify its potential in improving CRF.

This study has some limitations. First, we only retrieved English literature related to CRF from the SSCI and SCI-Expanded databases through the WoSCC. Unpublished articles and articles published in other languages were not included. Second, the literature search was limited to articles with “cancer-related fatigue” in their titles, abstracts, or keywords, potentially leading to the data being insufficiently comprehensive.

## 5 Conclusion

This study is the first and visualization analyses of cancer-related fatigue research published worldwide over the past two decades. The collaboration among researchers, institutions, and countries is limited. In addition, the results provides a comprehensive overview for global researchers, clinical practitioners, and decision-makers regarding the research focal points on risk factors, treatments, and potential mechanisms of cancer-related fatigue. Notably, the research findings indicate that hypoxia is a potential mechanism of cancer-related fatigue that merits further investigation. The HIF-related pathways could potentially serve as novel targets for the treatment of cancer-related fatigue.

## Data availability statement

The original contributions presented in the study are included in the article/**Supplementary Material**. Further inquiries can be directed to the corresponding authors.

## Author contributions

PL: Conceptualization, Data curation, Formal analysis, Investigation, Methodology, Supervision, Writing – original draft, Writing – review & editing. QW: Data curation, Formal analysis, Investigation, Methodology, Validation, Writing – original draft. LF: Conceptualization, Supervision, Writing – review & editing. ZD: Conceptualization, Funding acquisition, Supervision, Writing – review & editing. WF: Conceptualization, Methodology, Writing – review & editing.
